# Evolution and metabolite signaling of fumarases (class I and II): origin, function, and subcellular targeting

**DOI:** 10.1093/molbev/msag240

**Published:** 2026-10-12

**Authors:** Ophir Miller, Dror Jacobi, Liran Carmel, Ophry Pines

**Affiliations:** School of Computer Science and Engineering and the Faculty of Medicine, The Hebrew University of Jerusalem, Jerusalem 9190401, Israel; School of Computer Science and Engineering and the Faculty of Medicine, The Hebrew University of Jerusalem, Jerusalem 9190401, Israel; Department of Genetics, The Alexander Silberman Institute of Life Sciences, Faculty of Science, The Hebrew University of Jerusalem, Jerusalem 9190401, Israel; Department of Microbiology and Molecular Genetics, IMRIC, Faculty of Medicine, The Hebrew University of Jerusalem, Jerusalem 9112102, Israel

**Keywords:** fumarase, horizontal gene transfer, mitochondrial endosymbiosis, dual targeting, DNA damage response, metabolic signaling

## Abstract

This study explores the evolutionary origins of class I and II fumarases across the tree of life, focusing on the prevalence and phylogenetic patterns of their enzymes. Using a dataset of ∼21,000 complete proteomes and MMseqs2 easy-search against known fumarase sequences, we identified representative sequences for each class and constructed phylogenetic trees.

Class I fumarases are found primarily in bacteria and in several eukaryotic groups, including Protozoa, SAR, and algae, with absence in fungi and archaea and low presence in plants and animals. Their patchy distribution and absence in archaea suggest horizontal gene transfer from bacteria to eukaryotes. Class II fumarases are ubiquitously distributed across bacteria, archaea, and eukaryotes. Phylogenetic and genetic analyzes suggest a closer relationship between eukaryotic class II fumarases and bacterial homologs (particularly Alphaproteobacteria) compared to archaea. These results support the hypothesis that class II fumarase originated early on in bacteria and was then inherited by vertical gene transfer by eukaryotes.

Analysis of protein sequences and structures for both classes demonstrates high evolutionary conservation, consistent with their essential catalytic function. Fumarases, TCA cycle enzymes, have been implicated in DNA damage response in numerous organisms, in all domains of life. These enzymes have also been shown to be dual targeted to mitochondria and the cytosol/nucleus in eukaryotes. We have examined how these two functions are linked to the evolution of fumarases and concluded that the extramitochondrial functions and subcellular localization of fumarases were determined much later in evolution. We conclude that metabolic signaling and its specific metabolites may provide the driving force for the fumarases' dual function, dual targeting, and interaction with different entities in the cell.

## Introduction

Fumarase, also known as fumarate hydratase in multicellular eukaryotes, is a family of enzymes of the tricarboxylic acid (TCA) cycle, catalyzing the reversible hydration of fumarate to L-malate, an essential step in aerobic respiration (Puthan Veetil et al. 2012).

Structurally, fumarases fall into two unrelated classes (Woods et al. 1988; Ueda et al. 1991; Vugt-Lussenburg et al. 2013):

- Class I fumarases are iron-sulfur cluster-containing, oxygen-sensitive enzymes predominantly found in prokaryotes.
- Class II fumarases are iron-independent, heat-stable enzymes found across all domains of life, including eukaryotes.

In some organisms, such as *Escherichia coli*, both classes coexist (eg *fumA* and *fumB* are class I, and *fumC* is class II). Fumarases appear to have a complex evolutionary history involving gene duplication, interactions of the fumarase products with other proteins, and the acquisition of new cellular functions (Woods et al. 1988; Silas et al. 2021). In this regard, fumarases have been implicated in DNA damage response (DDR) in both bacteria and eukaryotes and in the production of organic acids destined for other metabolic pathways such as gluconeogenesis (Karniely and Pines 2005; Yogev et al. 2010). Another intriguing observation is that in eukaryotic cells, fumarase is notable for its presence in more than one organelle or subcellular compartment, a phenomenon termed dual targeting or dual localization, which is achieved by very different molecular mechanisms (Yogev et al. 2011).

Here, we examined whether the two classes were already present in the Last Universal Common Ancestor (LUCA). We found that while this is probable for class II, this does not appear to be the case for class I (Fox et al. 1980; Gerbod et al. 2001; Weiss et al. 2016; Berkemer et al. 2020; Moody et al. 2024). Moreover, we showed that mitochondrial class II enzymes in eukaryotes are more closely related to Alphaproteobacteria (the mitochondrial symbiont) than to Asgard archaea (the archaeal host) according to the “eocyte evolution theory” (Lake et al. 1984; Cox et al. 2008; Williams and Embley 2015; Rodrigues-Oliveira et al. 2023). Finally, the absence of class I fumarase in LUCA and in Asgard archaea implies that its eukaryotic origin is likely due to multiple horizontal gene transfer (HGT) events from bacteria. We have also examined how sequence patterns correlate with functions in DDR and protein localization in eukaryotes, using phylogenetic reconstruction, genetic distance metrics, and evolutionary conservation. In principle, we ask what was the driving force for the dual targeting and dual functions of the fumarases.

## Results

To explore the evolutionary landscape of fumarase enzymes, we first generated a comprehensive database of fumarase proteins across the entire tree of life. Initially, we sought to use the NIH's Database of Clusters of Orthologous Genes (COGs) (Tatusov et al. 1997), but the insufficient number of archaeal representatives (eg 99 for class II) and the absence of eukaryotic representatives led us to generate our own database. To this end, we probed the UniProt database, identifying fumarase proteins by running the MMseqs2 protein clustering tool (Steinegger and Söding 2017) against *E. coli* references for both class I and class II. Finally, we used stringent filtering criteria to generate our final dataset, spanning over 21,000 proteomes covering bacteria, archaea, and eukaryotes (see Methods). The choice of *E. coli* (of the Proteobacteria) class I and class II fumarases as probes for our analysis is due to the fact that *E. coli* is the best-annotated reference organism for fumarases (harboring both class II and class I), ensuring high-quality seed sequences. In addition, Alphaproteobacteria is a class of Proteobacteria that are the ancestors of the mitochondria via endosymbiosis by organisms related to Asgard archaea, thereby forming FECA (Schleper & Rodrigues-Oliveira 2026; Tobiasson et al. 2026).

### Class I fumarases show a patchy phylogenetic distribution

Class I fumarase displays a patchy and taxonomically restricted distribution, with no detectable presence in archaea, including the Asgard clade, which is considered the archaeal host lineage that gave rise to eukaryotes (Table 1). Within eukaryotes, class I fumarase is largely confined to SAR clade members, protozoans, algae, and a few lineages of animals and plants, and is absent from fungi. A phylogenetic tree constructed from representative class I sequences (Fig. 1) reveals a highly fragmented topology. Eukaryotic sequences do not form a monophyletic clade but instead spread among diverse bacterial lineages. This strongly argues against the presence of class I fumarases in LUCA, but rather points to the emergence of class I fumarase in bacteria, followed by a later introduction into several eukaryotic groups via multiple, independent HGT events.

**Figure 1:**
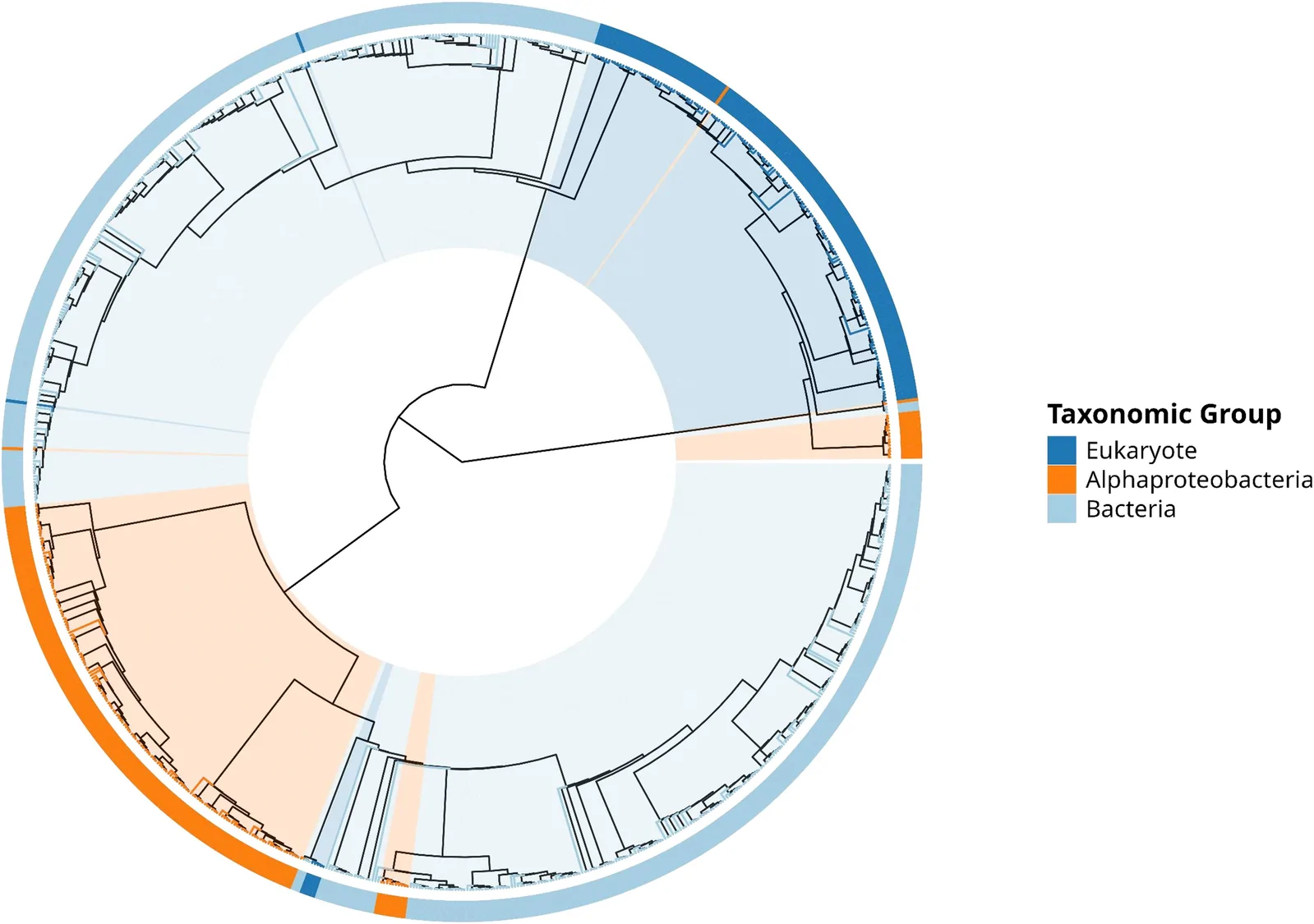
Phylogenetic tree of class I fumarase sequences. A maximum-likelihood tree based on representative class I fumarase sequences from across bacteria, archaea, and eukaryotes. Sequences are colored by domain. Eukaryotic sequences do not form a monophyletic group and instead cluster with diverse bacterial lineages. This fragmented tree structure supports multiple independent HGTs from bacteria into different eukaryotic lineages. Median bootstrap support is 99.

**Table 1:** Taxonomic distribution of class I fumarase across domains of life.

Taxonomic group	Proteomes	Harboring class l	Percentage harboring [%]
**All**	21,466	4,166	19.41
**Alphaproteobacteria**	2,484	627	25.24
**Other bacteria**	14,913	3,302	22.14
**Archaea**	632	0	0.00
**Eukaryota**	3,437	237	6.90
**Fungi**	1,494	0	0.00
**Animals**	1,277	81	6.34
**Plants**	416	19	4.57
**Protozoa**	63	37	58.73
**Algae**	230	115	50.00
**SAR**	166	90	54.22

### Class II fumarases seem to predate the formation of the 3 domains of life

Class II fumarases display a much broader and more continuous phylogenetic distribution. They are found in many archaeal, bacterial (including Alphaproteobacteria), and most eukaryotic lineages examined (Table 2). A phylogenetic tree constructed from class II sequences (Fig. 2) reveals largely coherent clade topologies, with bacterial lineages at the base. This widespread presence indicates that class II fumarase was already present in LUCA, and predates the divergence of the three domains of life (Singer et al. 2017; Silas et al. 2021). They therefore display a vertical mode of inheritance, or vertical gene transfer (VGT), reflected by a phylogenetic distribution that closely mirrors organismal phylogeny, lacking evidence for widespread HGT or domain-specific innovations.

**Figure 2:**
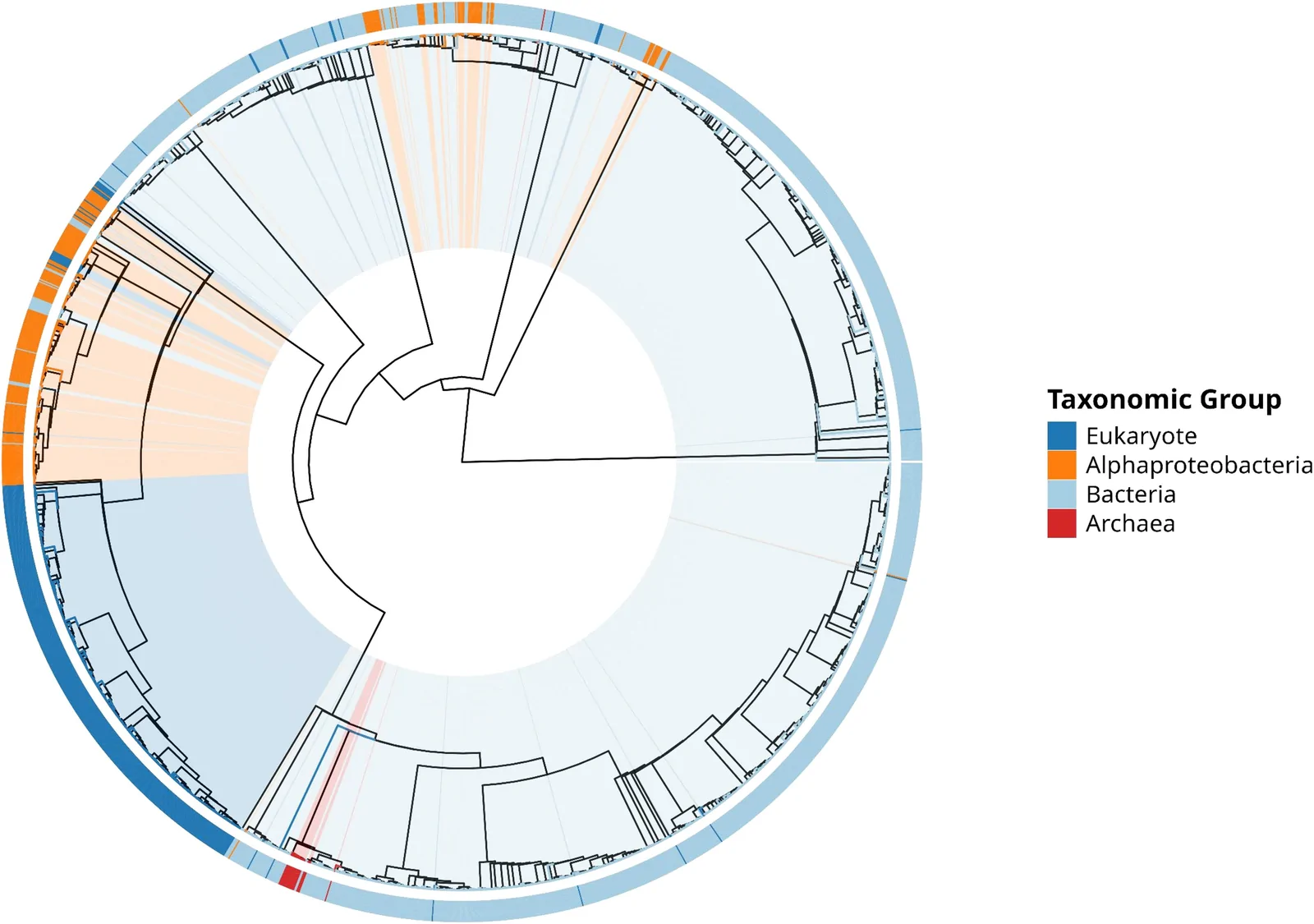
Phylogenetic tree of class II fumarase sequences. A maximum-likelihood tree based on representative class II fumarase sequences from across bacteria, archaea, and eukaryotes. Sequences are colored by phylum. Eukaryotic sequences cluster closely with Alphaproteobacteria, suggesting a mitochondrial origin. Median bootstrap support is 98.

**Table 2:** Taxonomic distribution of class II fumarase across domains of life.

Taxonomic group	Proteomes	Harboring class II	Percentage harboring [%]
**All**	21,466	17,183	80.05
**Alphaproteobacteria**	2,484	2,352	94.69
**Other bacteria**	14,913	11,389	76.37
**Archaea**	632	321	50.79
**Eukaryota**	3,437	3,121	90.81
**Fungi**	1,494	1,419	94.98
**Animals**	1,277	1,190	93.19
**Plants**	416	382	91.83
**Protozoa**	63	20	31.75
**Algae**	230	145	63.04
**SAR**	166	92	55.42

In eukaryotes, class II fumarase appears to have been retained from the mitochondrial endosymbiont—ie the First Eukaryotic Common Ancestor (FECA)—as the eukaryotic sequences cluster closely with those from Alphaproteobacteria (Fig. 2). In other words, the Alphaproteobacterial-like class II fumarase was likely inherited from the mitochondrial ancestor and retained in LECA (Last Eukaryotic Common Ancestor) (Singer et al. 2017). Worth mentioning is that a group of eukaryotic protozoans (*Giardia intestinalis*, *Trichomonas vaginalis*, and *Entamoeba histolytica*) may have gone through an HGT event of class II fumarases (Fig. 2).

To further resolve the evolutionary relationship of fumarase homologs across domains, we computed pairwise sequence identity among all sequences and grouped them by the three domains of life (Fig. 3). Eukaryotic sequences were found to be more similar to Alphaproteobacterial than to archaeal counterparts, with a median sequence identity of 62.50% compared to 50.50%. This difference was statistically significant (*P* < 2.2e-16, Mann-Whitney U test), affirming a bacterial origin for the eukaryotic class II fumarase, and more specifically, from the Alphaproteobacterial symbiont.

**Figure 3:**
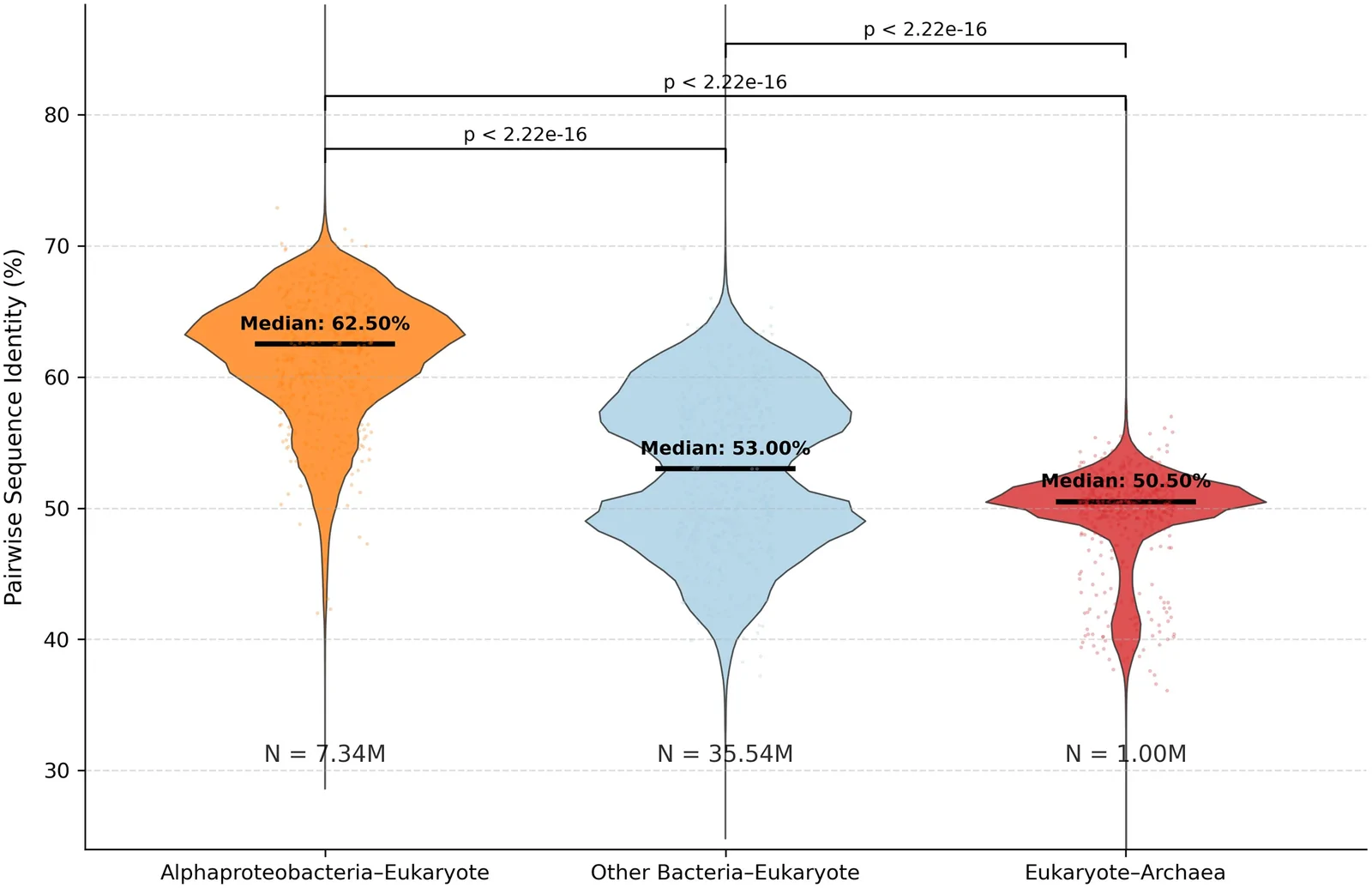
Sequence identity between eukaryotic fumarases and other domains. Pairwise identity (%) of class II fumarase sequences between eukaryotes and Alphaproteobacteria, other bacteria, or archaea. Eukaryote-Alphaproteobacteria pairs show the highest median identity (62.50%), followed by other bacteria (53.00%), and archaea (50.50%). All differences are highly significant (*P* < 2.2e-16, Mann-Whitney *U* test), supporting a bacterial—specifically Alphaproteobacterial—origin of eukaryotic class II fumarases.

To complement these analyzes, we embedded all protein sequences from both classes using the Evolutionary Scale Modeling (ESM-2) language model (Lin et al. 2023), which captures both functional and structural properties. For each protein, we calculated the pooled-mean embedding vector by averaging across all residue-level embeddings. We then applied t-SNE (van der Maaten and Hinton 2008) to project the high-dimensional embeddings into two dimensions, allowing visualization of sequence relationships within each class.

For class I (Fig. 4a), eukaryotic sequences largely clustered with Alphaproteobacteria, consistent with a mitochondrial/endosymbiotic origin, while a subset of scattered eukaryotic proteins may reflect additional, more recent HGT events. For class II (Fig. 4b), archaeal sequences formed a distinct cluster separated from Bacteria and Eukaryota, supporting VGT within the domain, whereas the eukaryotic and Alphaproteobacterial sequences formed a continuous gradient, indicating closer evolutionary proximity consistent with a shared mitochondrial origin.

**Figure 4:**
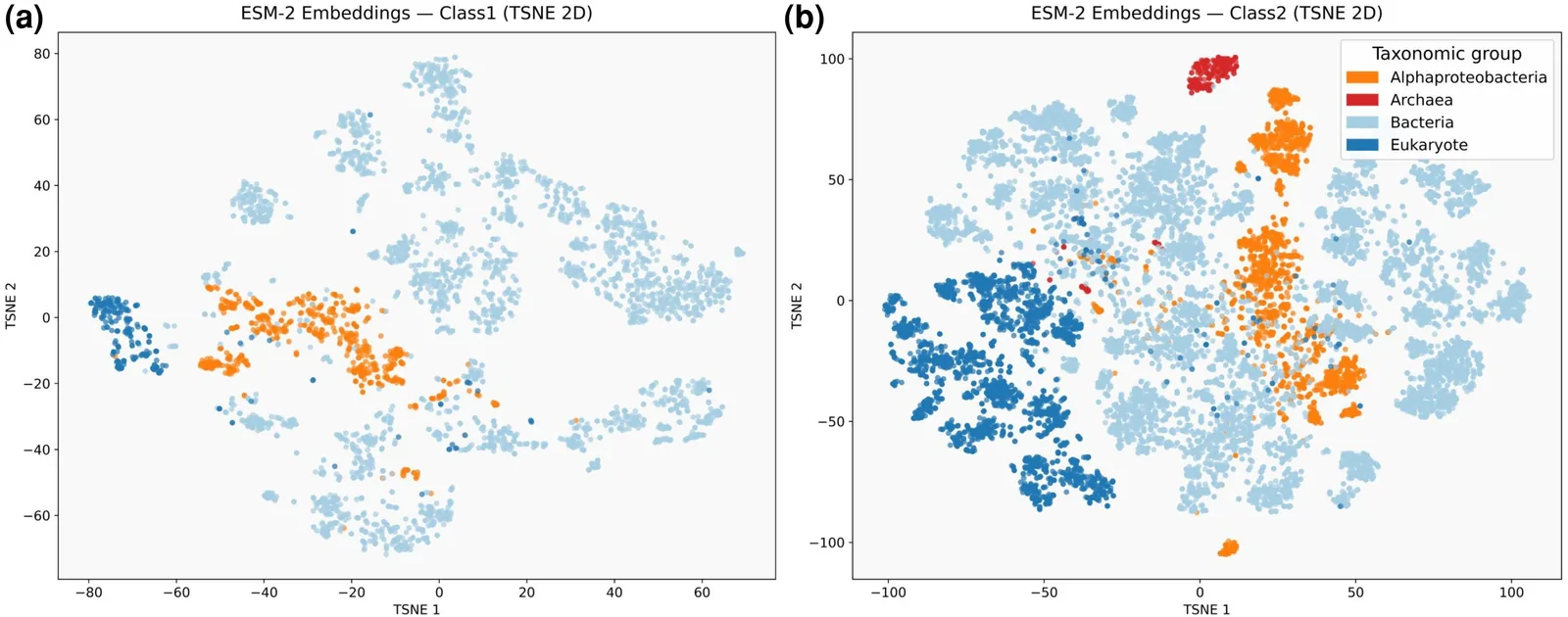
t-SNE projections of sequence embeddings. Visualization of protein sequences based on their mean ESM embeddings using t-distributed stochastic neighbor embedding (t-SNE). Each point represents a single protein. a) Class I eukaryotic fumarases generally cluster with the Alphaproteobacteria, with some eukaryotic proteins scattered across other locations, supporting recent HGT events. b) Class II archaeal fumarases form a separate cluster, away from the other domains, while the eukaryotic and Alphaproteobacteria groups form a continuum, supporting VGT.

These embedding-based patterns complement the phylogenetic tree topology (Figs. 1 and 2) and provide independent support for the differing evolutionary histories of class I and class II fumarases across the three domains of life.

### Both class I and class II show high sequence conservation, particularly at functional residues

Analysis of multiple sequence alignments demonstrates that both fumarase classes are under strong selective pressure to maintain their essential catalytic functions, resulting in high evolutionary conservation (Fig. 5). To quantify this, a Shannon entropy-based score was computed for each position, yielding mean scores of 0.987 bits for class I and 1.498 bits for class II fumarases (Capra and Singh 2007). To contextualize these values, we compared them to two baseline families: the highly conserved 50S ribosomal protein L1 (RplA), representing a standard for high conservation (mean entropy 1.499), and the divergent Major Facilitator Superfamily (MFS) transporters (mean entropy 3.210). Notably, class II fumarases exhibit conservation levels comparable to RplA (22.06% vs 22.65% highly conserved positions), while class I fumarases are significantly more conserved (41.06% highly conserved positions). Functional residues were nearly invariant, with mean site entropies of 0.000 for class I and 0.010 for class II (Weaver and Banaszak 1996; Mechaly et al. 2012; Ribeiro et al. 2018; Bateman et al. 2021). In this scoring system, values range from 0 (perfect conservation) to approximately 4.32 bits (maximum variability). This conservation was calculated using the full-length reference sequences of *E. coli* strain K-12's class I (UniProt: P0AC33, 548 residues) and class II (UniProt: P05042, 467 residues) enzymes.

**Figure 5:**
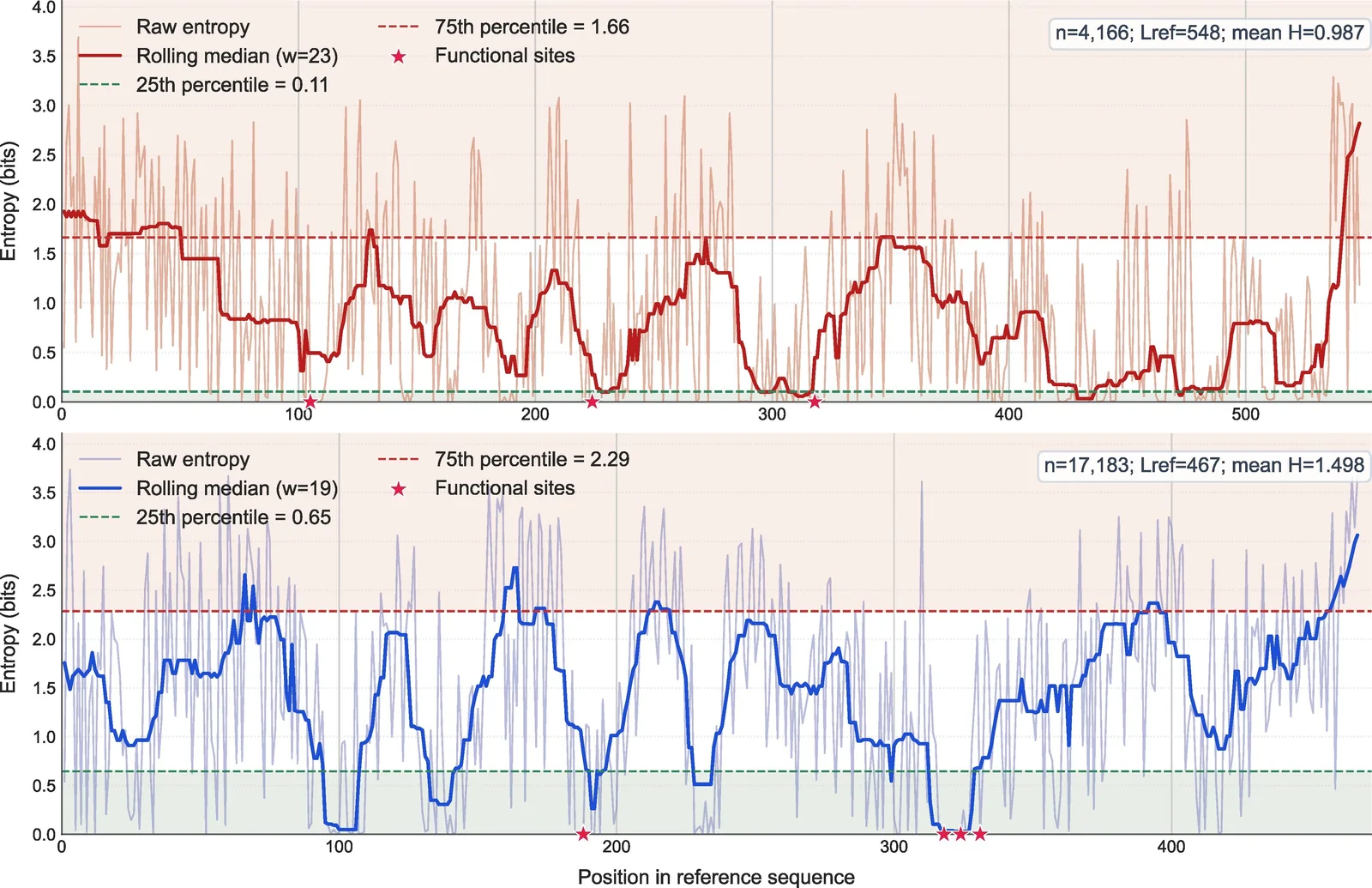
Conservation of class I and II fumarases with catalytic sites. a) Class I and b) class II conservation scores across E. coli reference sequences (P0AC33 and P05042, respectively). Red stars mark catalytic and substrate-binding residues (based on PDB annotations). Both classes show high conservation (low score) at functional sites.

In class I fumarase, key residues such as Cys105, Cys224, and Cys318 coordinate the [4Fe–4S] cluster essential for catalytic activity (Vugt-Lussenburg et al. 2013). In class II, critical active-site residues include His188, Ser318, Lys324, and Glu331, which directly participate in substrate turnover and stabilization of the transition state. Additionally, residues in close spatial proximity to these sites also display elevated conservation. This pattern highlights the strong evolutionary constraints acting on the catalytic cores of both fumarase classes (Wang et al. 2020; Yip et al. 2021).

This high conservation extends beyond the direct catalytic sites to a distributed network of residues critical for overall enzymatic function (Fig. 6a, b). A significant portion of these invariant residues constitutes the enzyme's structural scaffold, forming a stable hydrophobic core essential for global protein integrity and for maintaining the precise orientation and electrostatic environment of the active site. Furthermore, examination of the assembled structure for the functional tetramer confirms that other highly conserved patches are located at the subunit interfaces (Fig. 6c), mediating the specific protein-protein interactions required for the assembly and stability of the final quaternary structure. Thus, selective pressure acts not only on the active site itself but also on a widespread network of residues required for structural integrity and oligomeric assembly.

**Figure 6:**
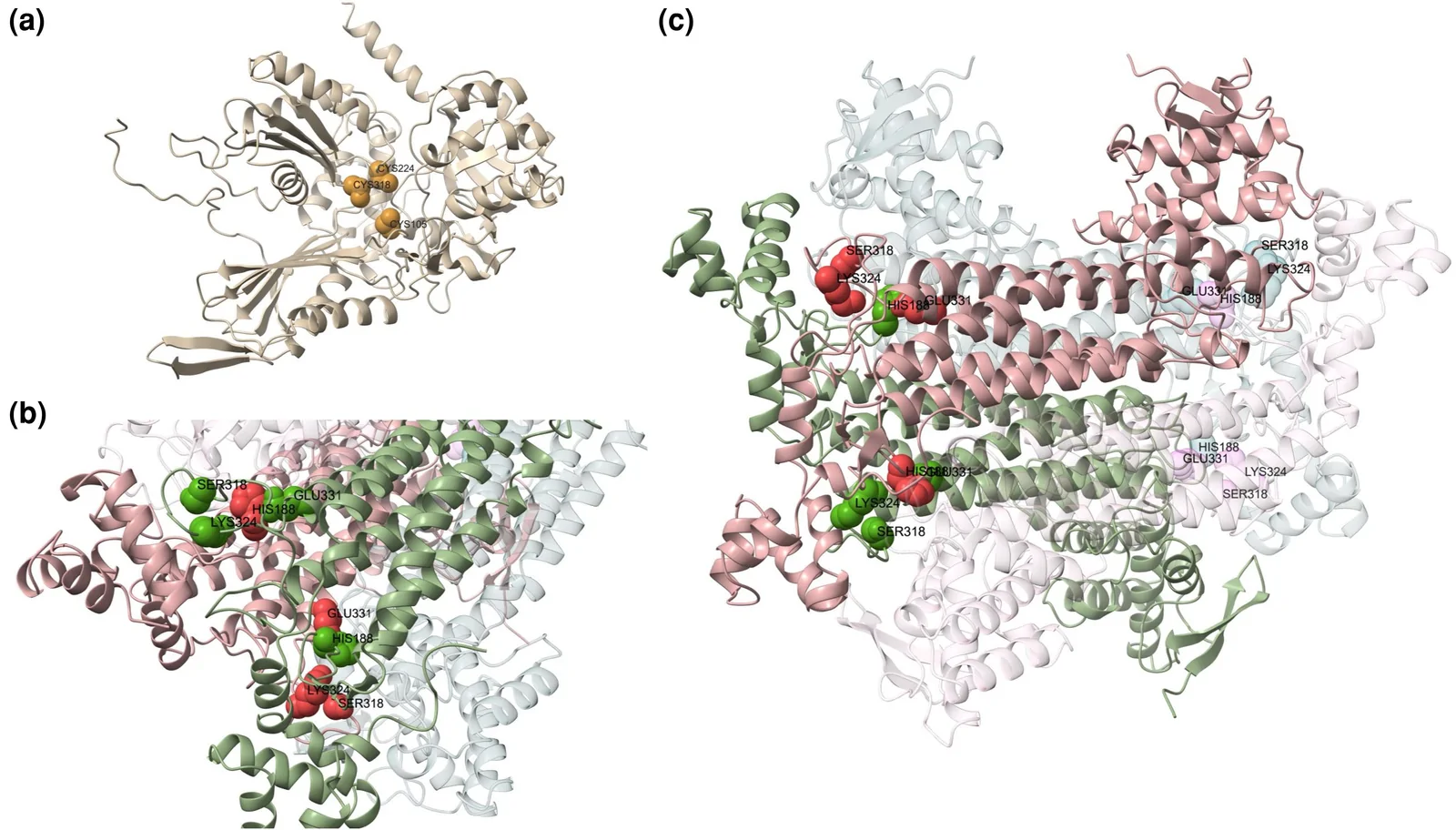
Structural conservation of fumarase class I and II. a) Class I fumarase (FumA): Structural representation of the E. coli reference (P0AC33). Residues (Cys105, Cys224, Cys318) coordinating the [4Fe-4S] cluster are shown as spheres. b) Class II fumarase (FumC): Structural representation of the E. coli reference (P05042), with subunits colored by chain and functional residues shown as spheres colored according to their originating chain. c) Class II tetramer: full view, with each subunit colored by chain to distinguish individual monomers.

### Structural relationships mirror sequence relationships

To further obtain structural insights into these evolutionary processes, we built for each fumarase class, a phylogenetic tree based on 3Di-encoded structural data. Class I fumarases contain an oxygen-sensitive [4Fe–4S] cluster and typically function as homodimers. These enzymes exhibit greater structural variability (Zubimendi et al. 2018), aligning with their patchier distribution and supporting the notion that they originated via multiple HGT events. This is consistent with our structural phylogeny (Fig. 7a), which shows class I fumarases forming scattered, taxonomically mixed clades without a clear domain-wide inheritance pattern.

**Figure 7:**
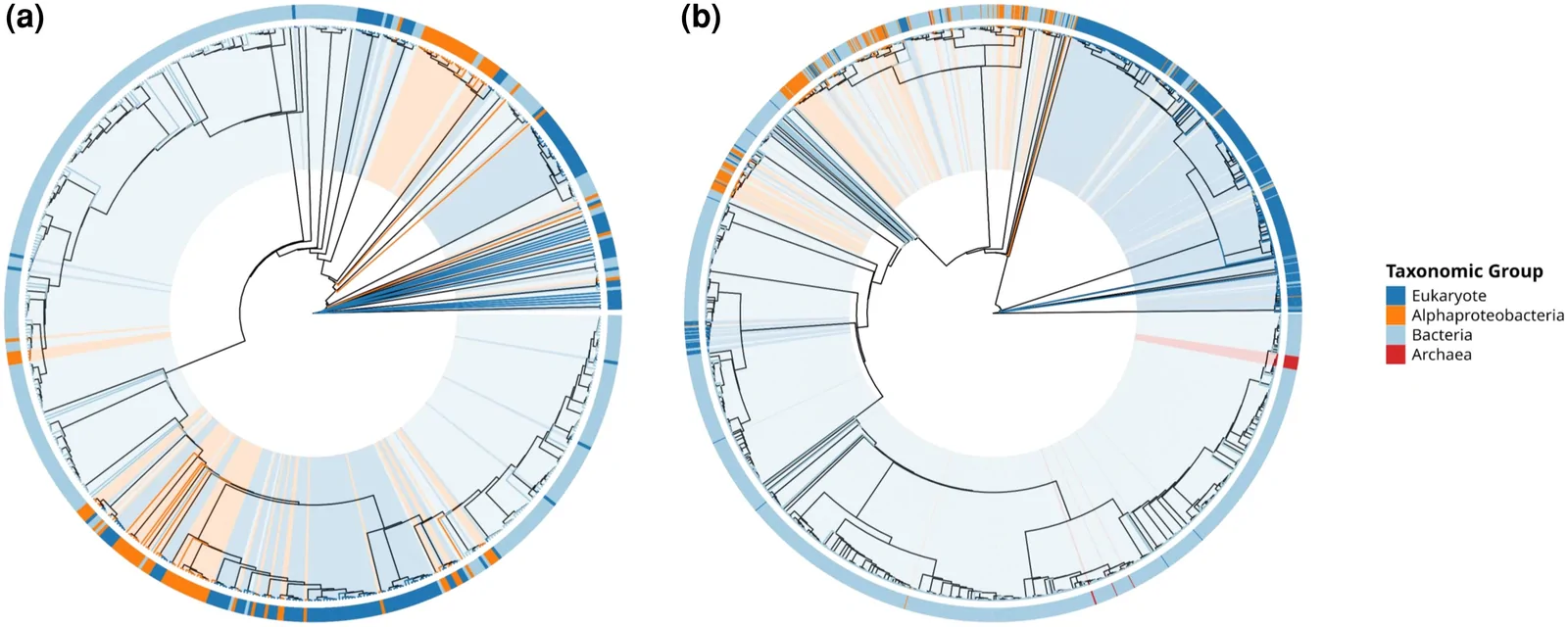
Phylogenetic distribution of class I and II fumarase based on ProstT5-derived 3di structural encodings. Circular phylogenetic tree reconstructed from 3Di sequences generated directly from amino acids using the ProstT5 encoder-decoder model. a) Class I fumarases show a scattered, patchy distribution and a mixture of distant taxa, supporting the hypothesis of multiple HGT events. b) Class II fumarases reveal a continuous and coherent topology, with eukaryotes clustering within Alphaproteobacteria, supporting vertical inheritance from the mitochondrial endosymbiont and their presence in LUCA. Similar to the sequence-based tree, we observe some later HGT events. Median bootstrap support for the structural trees is 83.

Class II fumarases are iron-independent enzymes that assemble into stable homotetramers, a structural feature conserved across bacteria, archaea, and eukaryotes (Woods et al. 1986). This tetrameric assembly contributes to their thermal stability and catalytic efficiency, potentially influencing their evolutionary persistence. Structural studies have demonstrated that the quaternary organization is critical for enzymatic function, with active sites formed at subunit interfaces (Weaver et al. 1995). The conservation of this tetrameric architecture across all domains of life supports the hypothesis of vertical inheritance (VGT) from LUCA. The structural classification within the aspartase/fumarase superfamily includes argininosuccinate lyases, enzymes that similarly form functionally critical multimeric assemblies (Yu and Howell 2000; Chen et al. 2019). Our structural phylogenetic tree supports this view (Fig. 7b), largely showing a coherent and continuous distribution of class II fumarases across domains.

Although the structural trees show somewhat lower median bootstrap support (83) compared to the sequence-based trees (98 and 99), the overall topological agreement between the two approaches strengthens our conclusions, as independent lines of evidence, and sequence and structure, converge on the same evolutionary relationships.

### Fumarases universally participate in DNA damage response (DDR)

The enzyme fumarase, in particular class II, has been associated with DDR in a wide variety of organisms, including eukaryotes, from humans and mice, through *C*. *elegans* and Arabidopsis *thaliana* to yeast (Saccharomyces *cerevisiae*) (Yogev et al. 2010; Leshets, Ramamurthy, et al. 2018; Leshets, Silas, et al. 2018; Giallongo et al. 2023; Álvarez-González and Sierra 2024). More recently, this has also been shown in prokaryotes, including *B. subtilis* (Gram-positive (Singer et al. 2017) and *E. coli* (Gram-negative (Silas et al. 2021). In these cases, fumarases can be shown by microscopic methods to be associated with nuclear/cellular DNA upon induced DNA damage (Table 3). It would be tempting to suggest that this function was the ancestral function of this protein, serving as the early driving force in its evolution. In that case, one would expect that the way fumarase and its activity are linked to the DDR would be conserved. In addition, one would expect that the proteins that fumarase and its organic acid products (fumaric and L-malic acids) interact with, during damage repair would be conserved. However, as shown in Table 3, the DDR targets of these organic acids are completely different, and so are the DDR factors bound to, or by, fumarases. While there is a clear pattern of fumarase participation in the DDR, the nature of the induced selective pressures should still be further elucidated.

**Table 3:** Fumarases and organic acids targeted to damaged DNA and DDR factors in different organisms.

Organism	Fumarase enzyme	Targeted to damaged DNA (Microscopy)	Active Fum/FH organic acid	DDR target of the organic acid	DDR factors bound to/by Fum/FH
***S. cerevisiae*** (Yeast)	Fum1 (class II)	Yes	Fumarate	Sae2	Sae2
***Homo sapiens*** (Humans)	FH (class II)	Yes	Fumarate	KDM2B/4B	PAR-Chains, DNA-PK
***B. subtilis*** (Gram + bacteria)	Fumbc (class II)	Yes	L-Malate	RecN	?
***E. coli*** (Gram- bacteria)	Fuma Fumb (class I)	Yes	Fumarate	AlkB	?

The association of fumarase with DNA, the organic acid involved and the targets of the signaling.

### Fumarases exhibit universal dual targeting patterns

The important conclusion from this section is that, even though fumarases are highly conserved, the mechanisms of their dual targeting in eukaryotes are not. In most eukaryotes examined, fumarase exhibits dual localization; it is found both in the mitochondria and the cytosol/nucleus. This includes humans (*Homo sapiens*), rats (*Rattus norvegicus*), *Caenorhabditis elegans*, *Arabidopsis thaliana*, *Saccharomyces cerevisiae*, and many others (Akiba et al. 1984; Carrie et al. 2009; Pracharoenwattana et al. 2010; Yogev et al. 2011). This dual targeting of echoforms (identical or nearly identical proteins situated in different subcellular locations) has a wide evolutionary distribution, but it was achieved via diverse mechanisms in different organisms (Table 4). In yeast (*S. cerevisiae)*, the single translation product of a single gene is co-translationally targeted to the mitochondrial translocation apparatus and processed, yet some of the molecules move back into the cytosol (Sass et al. 2001; Shoji et al. 2006). In rats, transcription of a single gene results in one type of transcription product, which then gives rise to two types of fumarases [one with and one without an mitochondrial targeting sequence{MTS}]. Thus, dual targeting of fumarase, in this case, is determined by the choice of the translation initiation codon by the ribosome (Tuboi et al. 1990). In human cells, transcription of the single gene results in two types of transcription products, one that contains the upstream ATG (translation initiation codon), and one that only contains the second ATG (Dik et al. 2016). If the ribosome initiates translation from the first AUG, we will get a product that contains the MTS, but if the ribosome initiates translation from the second AUG, we will get a shorter protein lacking the MTS. If the first AUG is missing from the shorter mRNA, only the short protein lacking the MTS will be made from this transcript. In *A. thaliana*, two different but  homologous genes encode two different translation products, one with and one without an MTS. The fact that these fumarases are dual localized in eukaryotic cells probably indicates dual function, such as the TCA cycle in mitochondria and DDR in the nucleus. Nevertheless, there are certainly other functions that these echoforms perform, such as participation in the production of cytosolic L-malate, the glyoxylate shunt, and gluconeogenesis (Pines et al. 1996; Regev-Rudzki et al. 2009). Thus, as pointed out above, even though fumarases are highly conserved, their mechanisms of dual targeting in eukaryotes are not.

**Table 4:** Dual targeting mechanisms of fumarases in eukaryotes.

Organism	Genes	Transcription products	Translation products	Dual localization mechanism	Level of regulation
***S. cerevisiae*** (Yeast)	1	1	1	Reverse translocation	Protein folding
***Rattus norvegicus*** (Rat)	1	1	2	One with and one without an MTS	Translation initiation
***Homo sapiens*** (Human)	1	Multiple	2	MTS on one, not on the other	Transcription initiation
***A. thaliana*** (Plant)	2	Multiple	2	MTS on one, not on the other	Genes

## Discussion

Fumarase (FH, fumarate hydratase), a key enzyme in the TCA cycle, exists in two structurally and evolutionarily distinct classes: class I and class II. While both catalyze the same reaction, their protein sequence, structure, and evolutionary history are completely different. In this regard, one may view class I versus class II as isoenzymes (or isozymes), which are variants of the same enzyme that catalyze the same chemical reaction but differ in amino acid sequence, physical properties, and kinetic parameters. The phylogenomic analysis reveals distinct evolutionary paths for fumarase classes I and II. Class I shows a fragmented and taxonomically narrow distribution, consistent with multiple HGT events from bacteria into specific eukaryotic lineages. Class II is widely conserved across all domains of life, supporting its presence in LUCA and its vertical inheritance into eukaryotes via the mitochondrial endosymbiont. In other words, the mitochondrial endosymbiont (harboring class II) was derived during evolution from Gram-negative Alphaproteobacteria and was then transferred by VGT (vertical inheritance) to eukaryotes.

While there is a clear pattern of fumarase participation in the DDR, the DDR targets of fumarase-related organic acids are completely different, and so are the DDR factors bound to or by fumarases. Thus, whereas fumarase seems to readily undergo exaptation into the DDR, this is achieved by totally different molecular components and mechanisms within different evolutionary lineages. The same is true regarding the dual localization of fumarases in eukaryotic cells, which is achieved by a different number of genes, transcription products, translation products, and molecular mechanisms in different eukaryotic lineages. If participation in the DDR or dual targeting of fumarase had occurred at a very early step of evolution (FECA or LECA), we would have expected greater mechanistic similarities across eukaryotes. We therefore assume that neither participation in the DDR nor the requirement for dual targeting occurred in FECA or LECA.

We propose that the driving force for fumarase-related functions throughout evolution is that fumarate/malate are not only metabolites for biosynthesis (building blocks) but also metabolic signaling molecules. Why then are specific metabolites required in different compartments of the cell? One clear possibility is that they are directly part of an enzymatic pathway required for the function of the cell, as are fumarate, succinate, and malate, which are required for the TCA cycle in mitochondria and prokaryotes. Another possibility is that metabolites are signaling molecules; in other words, they affect the activity and function of other molecules, primarily proteins. In this sense, fumarate and other metabolites affect the function of DDR (DNA damage response) systems in both eukaryotes and prokaryotes. While the participation of fumarate and fumarase in the TCA involves evolutionarily conserved proteins, this is not the case for the participation of fumarate and fumarase in the DDR, in which the metabolite and the enzyme interact with totally evolutionarily distinct proteins in different organisms. This is why we conclude that the driving force for the evolution of fumarase outside mitochondria is its requirement for the creation of signaling molecules such as fumarate and malate, and not for a specific DDR reaction. This explains why the enzyme and its organic acids must be involved in crucial activities such as the DDR, and why its enzymes are required both in and outside of mitochondria (dual localization). Thus, metabolic signaling and its specific metabolites provide the driving force for fumarase's dual function, dual targeting, and interaction with different entities in the cell.

## Methods

### Sequence retrieval and initial filtering

To investigate the distribution and evolutionary history of fumarase classes I and II across the tree of life, we first constructed a comprehensive fumarase sequence dataset. The initial query pool of sequences was the UniProt reference proteomes list (∼34,000 organisms), from which viral sequences were removed using a custom Python script based on taxonomy annotations, resulting in over 21,000 complete proteomes available in the UniProt Proteomes database (Bateman et al. 2021).

Individual proteome files were downloaded and searched in parallel using the MMseqs2 tool (Steinegger and Söding 2017). Reference sequences were compiled from the known UniProt *E. coli* fumarases: P05042 for class II (FumC) and P0AC33 for class I (FumA).

### Sequence identification and stringent filtering

Each proteome was scanned using MMseqs2 easy-search with a sensitivity of 7.5, running on a high-performance computing cluster using custom SLURM-based job scripts (Yoo et al. 2003). For each organism, search results were stored in .m8 files containing e-values, identity scores, bit scores, and sequence alignment details.

To assess fumarase presence, sequences were filtered according to the following criteria:

- e-value < 0.001.
- Identity ≥ 40%.
- Alignment covering ≥ 40% of the query length.

An additional filtering step used custom regex searching of protein descriptions to exclude known paralogs and mis-annotations, such as aspartate ammonia-lyase (AspA) for class II and triazine hydrolases or tartrate dehydratase subunits for class I. Organisms were considered to harbor a fumarase class if they contained at least one sequence meeting all stringent conditions.

### Taxonomic analysis

Organism metadata (taxonomic lineage, phylum, and kingdom) was parsed directly from UniProt's reference dataset and cross-referenced with filtered hits. We assigned each organism a binary presence/absence status, enabling analyses across taxonomic groups such as Archaea, Alphaproteobacteria, and Eukaryota. Hits were stored in FASTA files and summarized in taxonomic distribution tables.

### Alignment and conservation analysis

For both fumarase classes, sequences were aligned using MAFFT (Katoh and Standley 2013). The “auto” mode was employed to automatically select the optimal algorithm (eg FFT-NS-2 for large datasets). Conservation scores were calculated using Shannon entropy (Capra and Singh 2007; Roettger et al. 2009) across alignment columns, with scores mapped back to the *E. coli* reference positions (0 to 548 for class I; 0 to 467 for class II). Baseline sequences for the conserved RplA and divergent MFS transporter families were extracted for context comparison (Bateman et al. 2021).

Both classes’ sequences were clustered using MMseqs2 easy-cluster to remove redundant sequences, resulting in a non-redundant set of 898 representative sequences for class I and 6,006 for class II, which served as the basis for subsequent phylogenetic and structural embedding analyses.

From the alignment, phylogenetic trees were constructed using IQ-Tree2 (Minh et al. 2020) with the Q.pfam + R8 substitution model. The trees were visualized using the ggtree R package (Yu et al. 2017), with clade-level color coding by taxonomic group. Additional smaller trees were created for closer inspection and are available in the Mendeley Data repository (see Data availability).

Structural representations were obtained by translating protein sequences into 3Di sequences using the ProstT5 encoder-decoder model (Heinzinger et al. 2024). This sequence-to-sequence translation provides structural descriptors directly from amino acids without requiring explicit 3D models. Subsequently, those sequences were clustered using MMseqs2 easy-cluster, resulting in a non-redundant set of 581 representative sequences for class I and 2,329 for class II, following 3Di structural clustering. To ensure the structural signal was preserved during comparison, the 3Di sequences were aligned using MAFFT with the 3Di-optimized substitution matrix (van Kempen et al. 2024), implemented via the --aamatrix parameter. Finally, phylogenetic trees were constructed using IQ-TREE2, employing the PROST protein substitution model (Garg and Hochberg 2025), which is specifically designed to leverage the structural information embedded in ProstT5 representations.

Structural visualizations and molecular scenes were generated using ChimeraX (Meng et al. 2023). Because experimental structures were unavailable for class I, we utilized the AlphaFold model of the *E. coli* reference P0AC33 (Fleming et al. 2025). For class II, the AlphaFold model of P05042 was employed. To model the functional tetrameric state of class II, monomeric AlphaFold models were superimposed onto the subunits of the 1YFE PDB template (Weaver 2005). Catalytic residues—Cys105, Cys224, and Cys318 for class I, and His188, Ser318, Lys324, and Glu331 for class II—were highlighted using sphere representations to examine their spatial orientation within the core structure.

### Comparative genomics and embeddings

To quantify relatedness, we computed pairwise sequence distances using MMseqs2 identity scores. Statistics were calculated from an all-vs-all identity matrix for groups including Alphaproteobacteria, other bacteria, Archaea, and Eukaryota.

To better understand the relationship between eukaryotes and Alphaproteobacteria, we embedded all protein sequences from both classes using the ESM-2 model (esm2_t33_650M_UR50D). For each sequence, we computed the mean-pooling embedding vector by averaging across all residue embeddings. We then applied t-SNE (and UMAP) to visualize the resulting embeddings, coloring the points by taxonomic group. The projections recapitulated the expected phylogenetic patterns, further supporting the inferred relationships.

## Data Availability

All proteomic datasets were obtained from the UniProt reference proteomes database. Custom scripts for data processing, filtering (including regex-based description cleaning), and visualization are available in our GitHub repository: https://github.com/ophirmil12/FumaraseEvolution. Publicly available software includes MMseqs2, MAFFT, IQ-TREE2, ProstT5, and the ESM model series. No new experimental data were generated in this study. All supplementary information can be found under the following folder: https://doi.org/10.17632/4kvht65rtb.1.
